# Subcellular localization of fungal specialized metabolites

**DOI:** 10.1186/s40694-022-00140-z

**Published:** 2022-05-25

**Authors:** Elizabeth Skellam

**Affiliations:** Department of Chemistry and BioDiscovery Institute, University of North Texas, 1155 Union Circle, Denton, TX 76201 USA

**Keywords:** Subcellular compartmentalization, Fungal specialized metabolites, Fungal secondary metabolites, Fungal biosynthesis, Fungal sub-cellular localization, Fungal biosynthetic enzymes

## Abstract

Fungal specialized metabolites play an important role in the environment and have impacted human health and survival significantly. These specialized metabolites are often the end product of a series of sequential and collaborating biosynthetic enzymes that reside within different subcellular compartments. A wide variety of methods have been developed to understand fungal specialized metabolite biosynthesis in terms of the chemical conversions and the biosynthetic enzymes required, however there are far fewer studies elucidating the compartmentalization of the same enzymes. This review illustrates the biosynthesis of specialized metabolites where the localization of all, or some, of the biosynthetic enzymes have been determined and describes the methods used to identify the sub-cellular localization.

## Introduction

**Fig. 1:**
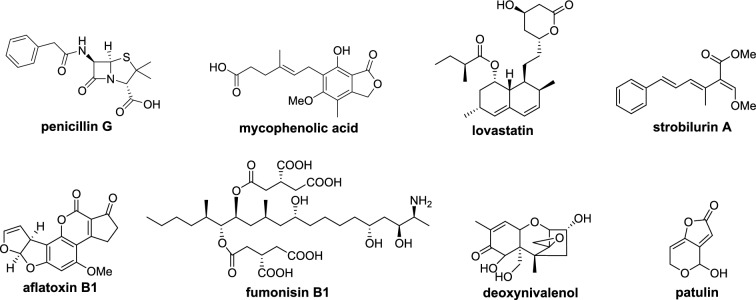
Examples of important fungal specialized metabolites

Specialized metabolites are small molecules found in plants, fungi, and bacteria, with a diverse array of molecular structures that vary considerably, even between closely related species. Specialized metabolites produced by fungi have specific ecological functions to aide in survival [1], and many have been adapted for medicinal or agricultural use [2] such as the antibiotic penicillin, the immunosuppressant mycophenolic acid, the cholesterol-lowering drug lovastatin, and the anti-fungal agent strobilurin (Fig. 1). Many fungal specialized metabolites, however, have a detrimental effect on human health and the environment [3] including the mycotoxins aflatoxin, fumonisin, deoxynivalenol, and patulin (Fig. 1).

**Scheme 1:**
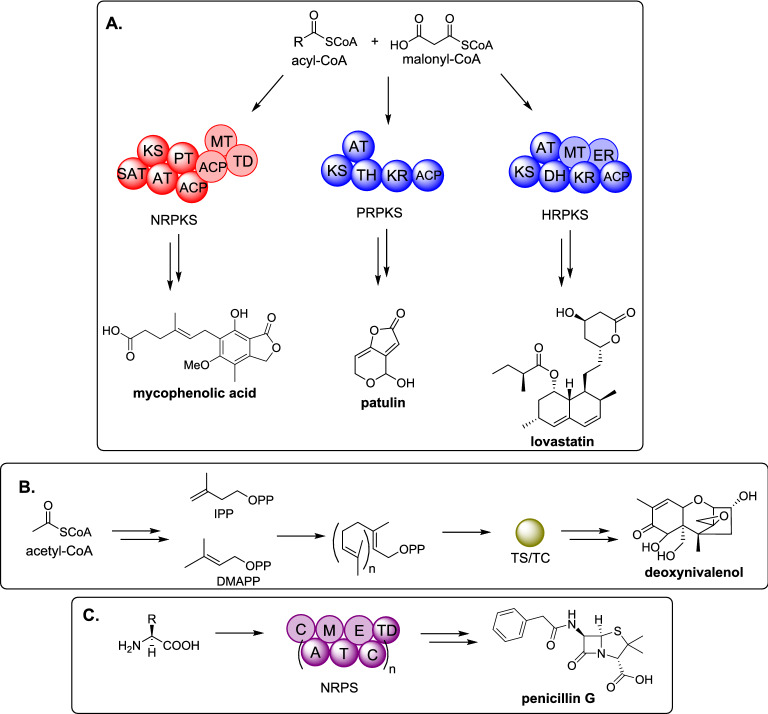
Overview of major classes of biosynthetic enzymes in fungi. **A** Polyketide biosynthesis; **B** terpenoid biosynthesis; **C** non-ribosomal peptide biosynthesis. SAT : starter unit: acyl-carrier protein transacylase; KS: ketosynthase; AT: acyltransferase; PT: product template; ACP: acyl carrier protein; MT: methyltransferase; TD: terminal domain; TH: thiolation; KR: ketoreductase; ER: enoylreductase; TS / TC: terpene synthase / terpene cyclase; C: condensation; A: adenylaton; M: methylation; T: thiolation; E: epimerase. Catalytical domains that are shown faded may or may not be present

Specialized metabolites are classified according to their biosynthetic origin and the major classes are: polyketides, terpenes, and non-ribosomal peptides; however molecules with mixed biosynthetic origin are common, including: meroterpenoids, polyketide/non-ribosomal peptides, and alkaloids. Polyketides are synthesized by polyketide synthases (PKS) from acyl-CoA substrates, most commonly acetyl-CoA and malonyl-CoA, although other small organic acids can be utilized [4]. In fungi, PKS enzymes can be further classified into non-reducing (NR) PKS, partially reducing (PR) PKS, and highly reducing (HR) PKS which vary according to the catalytic domains present, and the extent of reduction observed in the final polyketide product (Scheme 1A) [5, 6]. Terpenes are synthesized by terpene synthases (TS) (also known as terpene cyclases (TC)), from the head-to-tail condensation of isopentenyl pyrophosphate (IPP) and dimethylallyl pyrophosphate (DMAPP) (Scheme 1B) [7]. Non-ribosomal peptides are synthesized by non-ribosomal peptide synthetases (NRPS) that condense proteinogenic and non-proteinogenic amino acids to generate diverse peptides (Scheme 1C) [8]. Polyketide/non-ribosomal peptides are often synthesized by PKS and NRPS hybrids known as polyketide synthases / non-ribosomal peptide synthetase (PKS-NRPS) [9].

Typically, the final specialized metabolite observed has been modified or diversified by additional tailoring enzymes. The major classes of tailoring enzymes include: cytochrome P450 monooxygenases (P450), oxidases (OX), oxidoreductases (OXR), reductases (Red), hydrolases (HYD), dehydratases (DH), cyclases (CYC), methyltransferases (MT), and prenyltransferases (PT), although many additional types have been identified. Furthermore, there are increasing reports of protein-protein interactions between megasynth(et)ases (e.g. PKS and PKS-NRPS) and accessory and / or tailoring enzymes [10]. Additional, and often essential, enzymes required to efficiently regulate and excrete specialized metabolites include transcription factors (TF), transporters, and self-resistance proteins [1].

For most elucidated fungal biosynthetic pathways, the genes encoding the specialized metabolite are clustered together in the genome and co-regulated [1, 11]. Fungal biosynthetic gene clusters (BGCs) can readily be identified in sequenced genomes using bioinformatics tools such as fungiSMASH [12], and advances in molecular techniques such heterologous expression, gene disruption, and in vitro enzyme characterization enables the full elucidation of the pathway [13–15]. However, identifying the sub-cellular location of the individual enzymes within the native cell lags significantly behind. Understanding the sub-cellular localization, interactions, and transport of biosynthetic enzymes is useful for the efficient heterologous expression and engineering of BGCs in more amenable hosts for larger-scale production of medicinally or agriculturally important molecules [16]. This review highlights the progress that has been made in understanding where fungal specialized metabolites are synthesized within the cell and how the intermediates of these pathways are shuttled between locations and eventually exported.

## Subcellular localization of biosynthetic enzymes

**Fig. 2:**
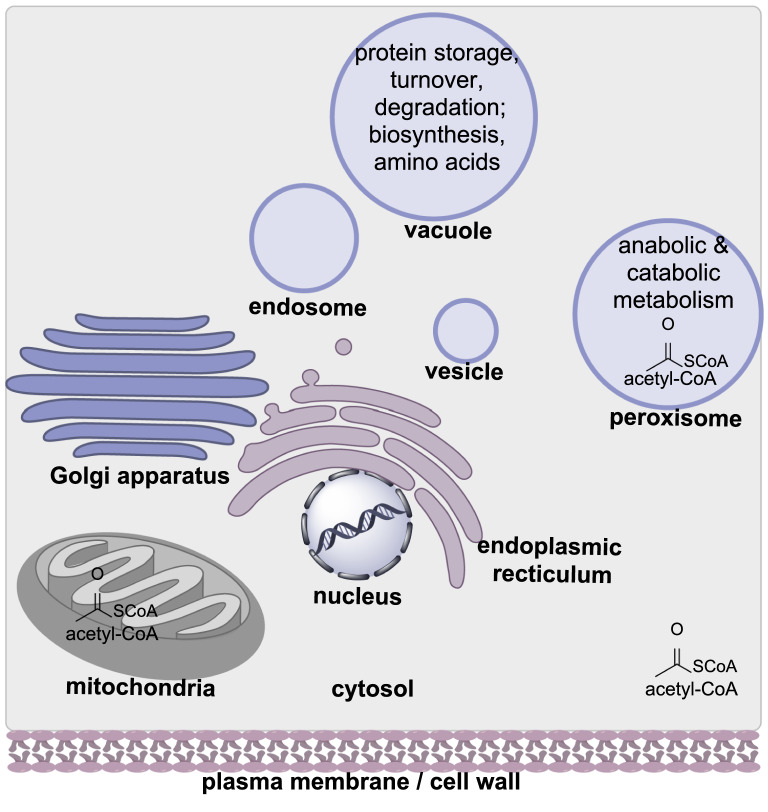
Cartoon schematic of a fungal cell showing the organelles most commonly associated with biosynthetic enzymes, precursors, and intermediates

Fungi have a complex cellular organization with numerous sub-compartments [17]. Fungal cells contain a nucleus, mitochondria, the endoplasmic reticulum, Golgi apparatus, and small membrane-bound organelles (Fig. 2). These small organelles can be further distinguished as vesicles, endosomes, vacuoles, and peroxisomes, each with variety of specific functions. Investigations into the function of the different organelles pertaining to specialized metabolism has indicated that synthesis and transport of metabolites, and associated biosynthetic enzymes, is a highly dynamic process [18]. Often multiple organelles are used, and evidence suggests that different organelles are used depending on whether biosynthetic enzymes are associated with early, middle, or late-stage biosynthetic modifications [19]. These organelles may also be referred to as “toxisomes” indicating that they contain potentially toxic pathway molecules [20, 21].

An overview of the experimentally established location of fungal biosynthetic enzymes are shown in Table 1. Enzymes required for synthesis of the carbon backbone of the molecule (e.g., PKS, NRPS, TS/TC) are highlighted in bold and do not appear to occur in a single organelle. For example, PKS enzymes have been identified in the cytosol, peroxisomes, and unidentified organelles. Furthermore, the biosynthesis of the same specialized metabolite may occur in different organelles depending on which fungal species is investigated e.g., melanin biosynthesis. And finally, the dynamic nature of specialized metabolism means that some biosynthetic enzymes are located in more than one organelle (e.g., the enzymes highlighted in italic in Table 1). Although a highly complex and understudied area of fungal specialized metabolism, this review is intended to collate the progress on identifying the subcellular localization of fungal biosynthetic enzymes and highlight potential common themes. For simplicity each investigated metabolite will be discussed separately according to its biosynthetic origin.

**Table 1:** The enzyme(s) required for synthesizing the carbon backbone for each specialized metabolite are highlighted in bold

Subcellular Localization of Biosynthetic Enzymes									
Specialized Metabolite	Biological Activity	Cytosol	Peroxisome	Nucleus	Endoplasmic Reticulum	Golgi	Vesicles / Endosomes / Vacuoles	Plasma Membrane	Cell Wall / Extra-cellular
Polyketides									
Aflatoxin	Mycotoxin	*AflD (KR)*, AflH (DH), AflM (OXR), *AflK (CYC*), AflP (MT)	**AflA (FAS), AflB (FAS), AflC (PKS),** HypC (OX)	*AflaR (TF)*, *AflaJ (TF)*	*AflK (CYC)*	*AflK (CYC)*	*AflD (KR)*, AflP (MT), *AflaR (TF)*, *AflaJ (TF)*		
Melanin	Photo- protectant / virulence factor	*BcBRN1 (Red)*, *BcBRN2 (Red)*,	**BcPKS12 (PKS),** **BcPKS13 (PKS),** BcYGH1 (HYD)				**Alb1 (PKS),** Agy1 (HYD), Arp1 (DH), Arp2 (Red)		Abr1 (OX), Abr2 (Lac),
							**WA (PKS)**		YA (Lac)
							*BcBRN1 (Red)*, *BcBRN2 (Red),*		BcSCD1 (DH), *BcBRN1 (Red)*, *BcBRN2 (Red)*,
Patulin	Mycotoxin	**PatK (PKS),** PatG (DCL), PatN (DH), PatF (U), PatD (DH), PatB (CE)		PatL (TF)	PatA (transporter), PatH (P450), PatI (P450)		PatJ (U), PatO (OXR), *PatC (MFS)*, *PatM (ABC)*, *PatE (OXR)*,	*PatC (MFS)*, *PatM (ABC)*	*PatE (OXR)*
Viriditoxin	Bacterial cell division inhibitor				VdtG (MFS)		**VdtA (PKS)**		
AK-Toxin	Mycotoxin		AKT1 (CA), AKT2 (HYD), AKT3 (ECH)						
Fumonisin	Mycotoxin (ceramide synthase inhibitor)	Fum3 (OX)		Fum21p (TF)			Fum8 (AMT), Fum17 (CS), Fum18 (CS), Cer1 (CS)		
A26771B	Antibiotic	BerkC (SDR)						BerkF (AT)	BerkD (OXR)
Terpenes									
Trichothecene	Mycotoxin	*Tri5 (TC)*, Hms1 (HMGS)		Tri6 (TF), Tri10 (TF)	Tri 1(P450), Tri4 (P450), Tri11 (P450), Tri14 (U), Red (P450R), Hmr1 (HMGR) *Tri5 (TC)*,				
Gibberellic acid	Phytohormone	**Cs/Ks (TC)**			HmgR (HMGR)		Ggs2 (GGPS)		
Paxilline	Mycotoxin		**PaxG (GGPS)**						
Aphidicolin	DNA polymerase inhibitor	**PbACS (TC),** PbGGS (GGPS)			PbP450-1 (P450), PbP450-2 (P450)				
Mixed									
Mycophenolic acid	Immunosuppressant agent	**MpaC’ (PKS),** MpaG’ (MT)	PbACL891 (ACL), MpaH’ (ACH)		MpaDE’ (P450 / HYD), MpaB’ (OX),	MpaA’ (PT)			
Peptides									
Penicillin	Antibiotic	**ACVS (NRPS)**, *IPNS (OX)*	*IPNS (OX)*, PenM (transporter), PaaT (transporter), IAT (AT), PCL (CoAL)				**ACVS (NRPS)**, PenV (transporter)		
Cephalosporin	Antibiotic		CefP (transporter), CefD1 (CoAL), CefD2 (epimerase), CefM (transporter)				*CefT*		*CefT*
Fumiquinazolines	Cytotoxic	FmqB (OXR), **FmqC (NRPS)**					**FmqA (NRPS),** FmqE (MFS)	FmqD (OXR)	
Cyclosporin	Immunosuppressant agent						**SimA (NRPS)** SimB (AlaR)		
Triacetylfusarinine C	Siderophore	EstB (HYD)	SidF (CoAT), SidH (CoAH), SidI (CoAL)						
Ferricrocin	Siderophore	SidL (AT)						Enb1 (Trans)	
Enterobactin	Siderophore						Sit1 (Trans)		
Ferroxamine B	Siderophore						Arn1p (Trans)		
Ferrichrome	Siderophore								
Amanitin	RNA polymerase II inhibitor						AbPOPB (POP)		

Abbreviations (alphabetical): ABC: ATP binding cassette transporter; AlaR: alanine racemase; AMT: aminotransferase; AT: acyltransferase; CA: carboxyl-activating enzyme; CE: carboxyesterase; CoAH: mevalonyl-CoA hydratase; CoAT: anhydromevalonyl-CoA transferase; CoAL: mevalonyl-CoA ligase; CS: ceramide synthase; CYC: cyclase; DCL: decarboxylase; DH: dehydratase; ECH: enoyl-CoA hydrolase; FAS: fatty acid synthase; GGPS: geranyl phosphate synthase; HMGS: HMG-CoA reductase; HMGS: HMG-CoA synthase; HYD: hydrolase; KR: ketoreductase; Lac: laccase; MFS: major facilitator superfamily transporter; MT: methyltransferase; NRPS: non-ribosomal peptide synthetase; OX: oxidase; OXR: oxidoreductase; P450: cytochrome P450 monooxygenase; PKS: polyketide synthase; POP: prolyl oligopeptidase; Red: reductase; SDR: short chain dehydratase / reductase; TC: terpene cyclase; TF: transcription factor; U: unknown

### Subcellular compartmentalization of polyketide biosynthesis

#### Aflatoxin biosynthesis

**Scheme 2:**
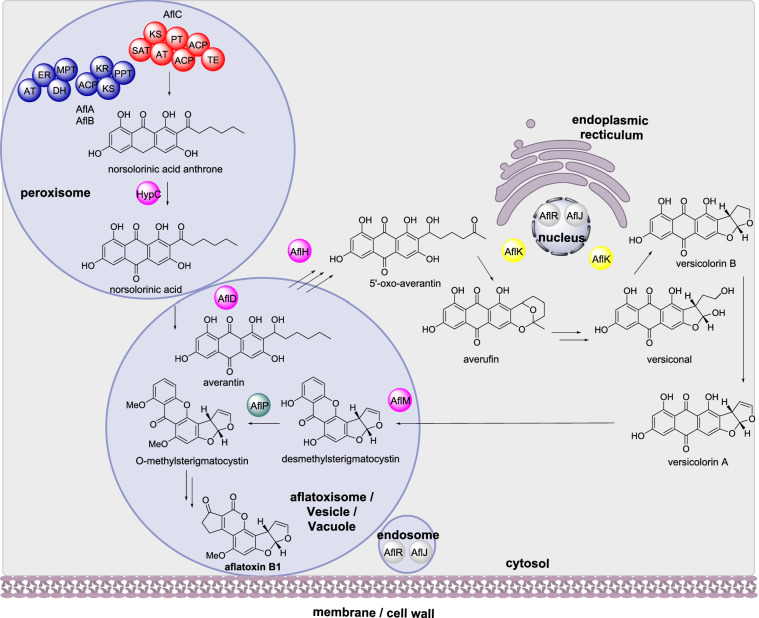
The sub-cellular biosynthesis of aflatoxin B1. Only biosynthetic enzymes with an established location are depicted

Aflatoxin is one of the most potent carcinogens known and is produced primarily by *Aspergillus parasiticus* and *Aspergillus flavus* [22]. The biosynthesis of aflatoxin requires over 25 genes which encode over 27 enzymatic activities. The BGC is regulated by the transcriptional regulator AflR and the transcriptional co-activator AflJ [23]. The first biosynthetic step is the synthesis of norsolorinic acid anthrone through the action of two fatty acid synthases (FAS; AflA and AflB) and a polyketide synthase (PKS; AflC), which form a protein complex (Scheme 2) [24, 25]. Norsolorinic acid anthrone is then oxidized into norsolorinic acid by HypC [26]. Further processing and modification of norsolorinic acid into aflatoxin is performed by a series of tailoring enzymes (Scheme 2). Modifications include: reduction by the NADPH-dependent ketoreductase (KR) AflD (also known as Nor-1) that converts norsolorinic acid to averantin [27]; oxidation by the alcohol dehydrogenase AflH that converts 5-hydroxy-averantin to 5’oxoaverantin [28]; and cyclization by the novel cyclase (CYC) AflK which converts 5’-oxoaverantin to averufin and subsequently versicolorin [28, 29]. In later steps, versicolorin A is converted to demethylsterigmatocystin by the NADPH-dependent reductase (Red) AflM (also known as Ver-1) [30] and sterigmatocystin is converted to O-methylsterigmatocystin by the methyltransferase (MT) AflP (also known as OmtA) [31].

The subcellular localization of many of the enzymes has been established using a variety of techniques including fluorescence protein fusions, transmission electron microscopy, and immunofluorescence microscopy. AflA, AflB, AflC and HypC are speculated to be located within peroxisomes due to the accumulation of norsolorinic acid in the peroxisomes [32]. Studying AflD fused to eGFP by confocal microscopy indicated that the enzyme is first synthesized in the cytoplasm and transported to vacuoles where it is active [33]. AflH was identified from the cytosolic fraction but its precise subcellular localization is unknown [28]. Similarly, AflK was identified from the cytosolic fragment of *A. parasiticus* initially and later shown by confocal laser scanning microscopy and immunogold transmission electron microscopy to be distributed in the cytoplasm and concentrated in structures that are ring-like and closely positioned on the outside surface of nuclei (Chiou et al. [34]). Examination of the protein sequence of AflK identified a signal peptide indicative of endoplasmic reticulum / Golgi localization [34]. Fusion of enhanced green fluorescence protein (eGFP) to AflM demonstrated that AflM is synthesized and localized in the cytoplasm and was found in the lumen of up to 80% of vacuoles [35]. Immunofluorescence microscopy determined that AflP is localized in the cytoplasm as well as vacuoles [36]. Using yellow fluorescence protein fused to AflJ indicated that AflJ localizes mostly in endosomes, and that AflR co-localizes with AflJ both in endosomes and nuclei [37].

#### Melanin biosynthesis

**Scheme 3:**
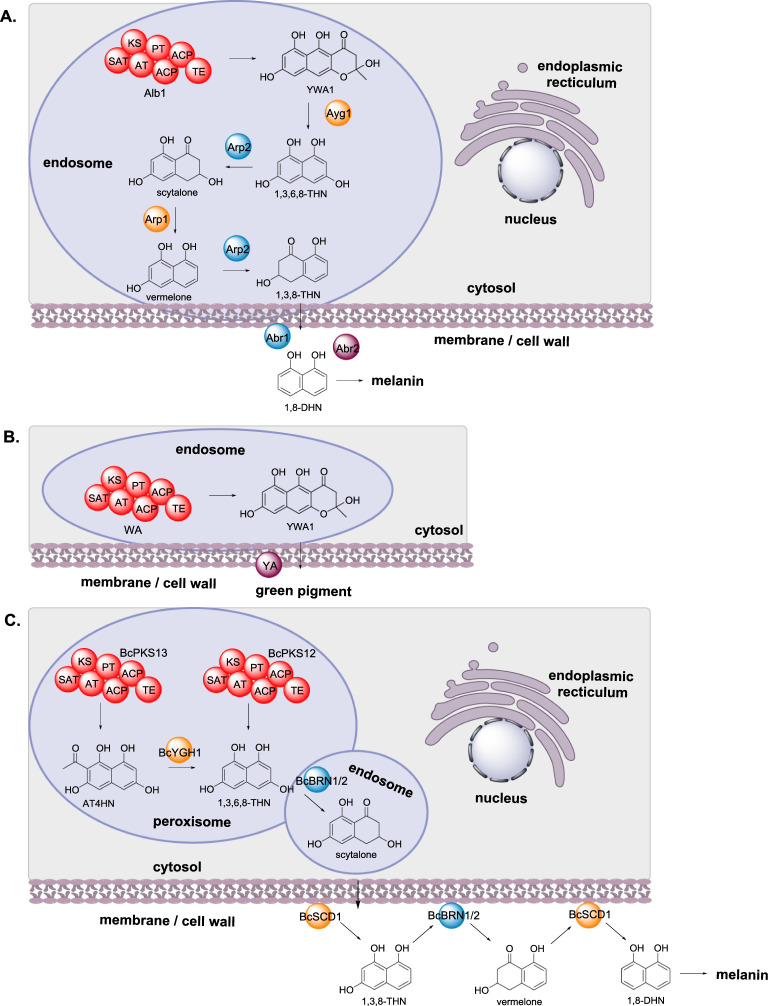
The sub-cellular biosynthesis of melanin in different fungi. **A** *A. fumigatus*. **B** *A. nidulans*. **C** *B. cinerea*

Fungal melanin is a pigmented biopolymer that protects its host from UV radiation, has an essential role in survival and infection, and is found as globular particles in fungal cell walls [38]. Most filamentous fungi synthesize melanin *via* a polyketide pathway, although the exact pathway has been demonstrated to differ between fungi. In *Aspergillus fumigatus*, biosynthesis of melanin requires at least six enzymes: the NRPKS Alb1 that synthesizes the polyketide YWA1 [39]; the chain shortening hydrolase (HYD) Ayg1 that converts YWA1 to 1,3,6,8-tetrahydroxynaphthalene (THN) [40, 41]; the reductase (Red) Arp2 that reduces 1,3,6,8-THN to scytalone; the scytalone dehydratase (DH) Arp1; the copper oxidase (OX) Abr1; and laccase (Lac) Abr2 (Scheme 3A). Arp1 dehydrates scytalone to produce 1,3,8-THN, and Arp2 acts a second time reducing 1,3,8-THN to vermelone. Vermelone is then oxidized by the copper oxidase Abr1 to 1,8-dihydroxynaphthalene and polymerized by the laccase Abr2 to melanin (Scheme 3A) [42, 43].

The localization of the melanin biosynthetic enzymes from *Aspergillus fumigatus* were investigated using fluorescence tags and specific organelle stains [19]. The PKS Alb1 was localized to endosomes, rather than being diffused through the cytoplasm as predicted bioinformatically. Furthermore, the additional three enzymes, Ayg1, Arp1, and Arp2, involved in early steps of the biosynthesis were also observed in endosomes, also in contrast to bioinformatics predictions. The two late-stage enzymes Abr1 and Abr2, predicted as secreted proteins, were found to accumulate at the cell wall [19].

*Aspergillus nidulans* also synthesizes melanin pigments utilizing a different pathway, where only two enzymes have been identified [44, 45]; the NRPKS WA and the laccase YA (Scheme 3B). Bioinformatics analysis predicts WA to be a cytosolic enzyme and YA a secreted enzyme, which indicates a clear separation of early-stage and late-stage melanin biosynthetic enzymes, common to many fungi [19]. However, their subcellular localization was determined to be in endosomes and at the cell wall respectively [19].

Investigation into the biosynthesis of melanin in *Botrytis cinerea* led to the observation that scytalone is secreted extracellularly and accumulation of this metabolite inhibits the growth of *B. cinerea* [46]. Similar to melanin biosynthesis in *A. fumigatus*, melanin biosynthesis in *B. cinerea* requires six biosynthetic enzymes: the PKS BcPKS12 (or BcPKS13 with the hydrolase BcYGH1); the reductases BcBRN1 and BcBRN2; and the dehydratase BcSCD1 (Scheme 3C) [47]. The subcellular location of the different melanin biosynthetic enzymes was determined *via* fusion with GFP in combination with organelle stains. The two PKS, BcPKS12 and BcPKS13, and the hydrolase BcYGH1 were demonstrated to be localized in peroxisomes, the two reductases BcBRN1 and BcBRN2 were detected within endosomes initially, and BcSCD1 is localized at the cell wall (Scheme 3C) [46]. The specific locations of BcBRN1 and BcBRN2 is further described in "Transient recruitment of enzymes and trafficking" section.

#### Patulin biosynthesis

**Scheme 4:**
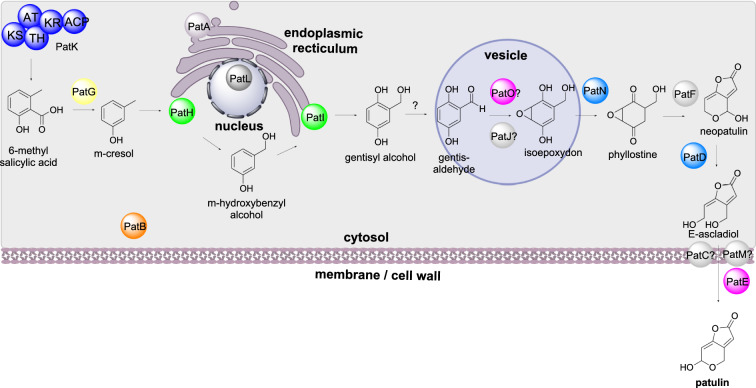
The sub-cellular biosynthesis of patulin. The final biosynthetic conversion occurs outside of the cell

Patulin is a polyketide-derived mycotoxin that is found in fruit and is produced mainly by *Penicillium expansum* [48]. The biosynthesis of patulin requires at least 10 steps and the BGC encodes 11 biosynthetic enzymes, a transcription factor, and three transporters [49]. PatK is a PRPKS that synthesizes 6-methylsalicylic acid that is then decarboxylated by the 6-methylsalicylic acid decarboxylase PatG, and sequentially hydroxylated by PatH and PatI, both P450s [50–52]. It is unknown how gentisyl alcohol is converted to isoepoxydon, however, the oxidase PatO and hypothetical protein (U) PatJ are proposed to be involved [53]. The dehydrogenase PatN converts isoepoxydon to phyllostine which is converted to neopatulin *via* the hypothetical protein PatF [53]. The dehydrogenase PatD converts neopatulin to E-ascladiol and then finally the action of the oxidoreductase PatE results in the synthesis of patulin (Scheme 4).

The localization of the enzymes responsible for biosynthesis for patulin were determined by fusing eGFP at their C-terminal [53]. Most biosynthetic enzymes (PatK, PatG, PatN, PatF, and PatD) were detected within the cytosol. PatB, a carboxyesterase, was also detected in the cytosol, however its precise biosynthetic role is unknown. The oxidoreductase PatE is localized to the cell wall and believed to be a secreted protein, whereas the two P450s PatH and PatI are localized to the endoplasmic reticulum. PatA, an acetate transporter, was also localized to the endoplasmic reticulum and the two transporters PatC and PatM were localized to the plasma membrane [53]. PatL is a Zn(II)2Cys6 binuclear cluster transcription factor and is localized in the cell nuclei [49].

#### Viriditoxin biosynthesis

**Scheme 5:**
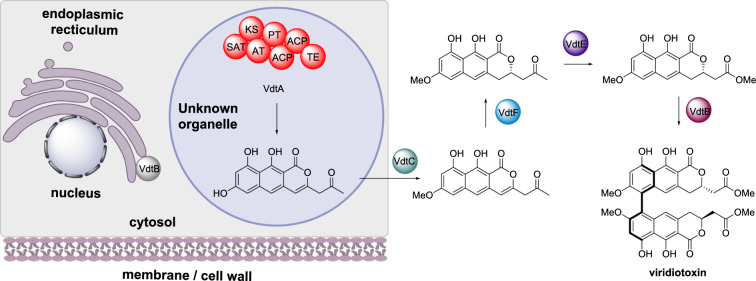
The partial sub-cellular biosynthesis of viriditoxin. The final steps of the biosynthesis are shown for completeness, however, the localization of the enzymes shown outside of the cartoon cell are unknown

Viriditoxin is a biaryl polyketide that exhibits antibiotic and cytotoxic bioactivities [54]. The biosynthesis was established in *Paecilomyces variotti* where the NRPKS VdtA synthesizes the polyketide backbone [55]. This is followed by methylation by VdtC, an O-methyltransferase, reduction by VdtF, oxygen insertion via VdtE, and dimerization catalyzed by the laccase VdtB. A transporter VdtG and regulator VdtR were also encoded by the biosynthetic gene cluster (Scheme 5). The subcellular localization of VdtA was investigated through fusion with GFP [55]. Fungal strains expressing this VdtA-GFP fusion protein displayed green fluorescence that was localized to small circular structures within the cells. These structures were deduced as not being peroxisomes, nuclei, or mitochondria [55]. The subcellular localization of the transporter VdtG was also investigated using GFP fusions and determined to be situated at the endoplasmic reticulum membrane, potentially within a toxisome.

#### AK-toxin biosynthesis

**Scheme 6:**
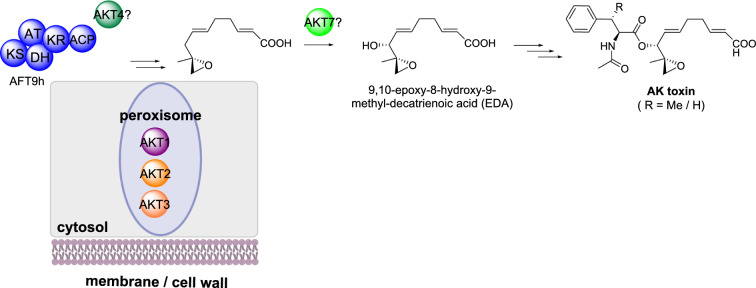
The partial sub-cellular biosynthesis of AK toxin. The general biosynthesis is shown for completeness, however, the localization of the enzymes shown outside of the cartoon cell are unknown

AK toxin is a polyketide-derived mycotoxin isolated from *Alternaria alternata* responsible for causing black spot of Japanese pears [56]. The polyketide portion, 9,10-epoxy-8-hydroxy-9-methyl-decatrienoic acid (EDA), present in several other toxins also isolated from *A. alternata* isolates, is proposed to arise from an unusual highly reducing polyketide synthase (HRPKS), a HMG-CoA synthase (HMGS) and a P450, at a minimum (Scheme 6) [57]. A BGC has been identified that encodes a PKS (AFT9h), a carboxyl-activating enzyme (AKT1), an α,β-hydrolase (AKT2), an enoyl-CoA hydratase (AKT3), a HMG-CoA synthase (AKT4), a P450 (AKT7) and a transcriptional regulator (AKTR), however the full sequence of biosynthetic steps remains to be elucidated [57–60].

Orthologs of the biosynthetic genes AKT1, AKT2, AKT3 are conserved in *A. alternata* strains that also produce toxins with an EDA core and therefore are believed to be required for the biosynthesis of this polyketide [61]. Analysis of the encoded enzyme sequence identified putative peroxisome tripeptide sequences in AKT1, AKT2 and AKT3. Localization of each enzyme to peroxisomes was confirmed by creating N-terminal GFP fusions that were expressed into *A. alternata* resulting in fluorescence localized to punctate organelles [61]. When the C-terminal tripeptide sequence was deleted, the resulting observed fluorescence was cytosolic [61].

#### Fumonisin biosynthesis

**Scheme 7:**
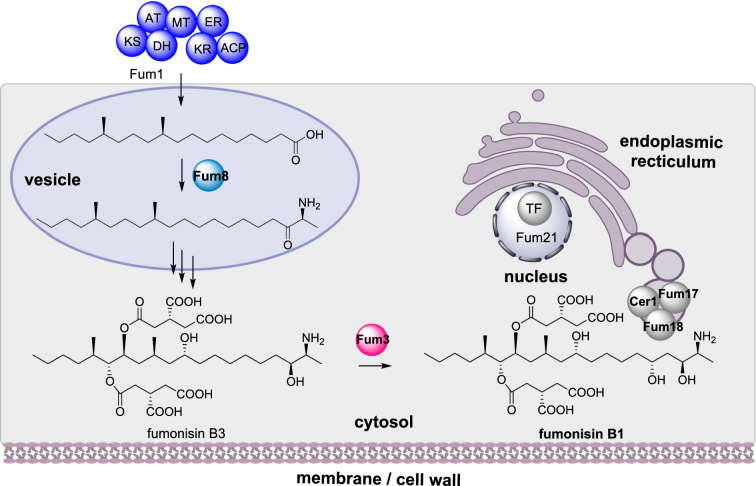
The sub-cellular biosynthesis of fumonisin B1. Other than the PKS Fum1, localization unknown, only biosynthetic enzymes with an established location are included in the cartoon cell

Fumonisins are polyketide-derived mycotoxins produced by *Fusarium* and *Aspergillus* sp. that contaminate corn [62]. Fumonisin B1 (FB1) is an inhibitor of sphingolipid biosynthesis, which leads to interference with eukaryotic cellular membranes, potentially leading to disease in humans and animals [63]. In *Fusarium verticillioides* the fumonisin BGC contains 16 genes including a HRPKS (Fum1) that synthesizes an octadecanoic acid precursor and the aminotransferase (Fum8) that condenses L-alanine with the polyketide (Scheme 7). Fum11, a tricarboxylate transporter; Fum10, an acyl-CoA synthetase; Fum7, a dehydrogenase; and Fum14 a bidomain NRPS; synthesize and transfer the sidechain to generate FB3 [64, 65]. Finally, the dioxygenase Fum3 adds a single hydroxyl group converting FB3 to FB1 [64].

Although the BGC has been extensively investigated, the functions of several genes (FUM15 – FUM19) remained unclear and the self-protection mechanism was unknown. Through gene disruption studies Fum19 was determined to be an ABC transporter that acts as repressor of the FUM BGC, and Fum17 and Fum18 were established as ceramide synthases which confer resistance to FB1 [66]. Furthermore, an additional three ceramide synthases (Cer1 – Cer3) were also found in *F. verticillioides* outside of the FUM BGC. To investigate the subcellular localization of Fum3, Fum8, Fum17, Fum18, and Cer1 were fused to different combinations of green, red, and blue fluorescent proteins. Fum17, Fum18 and Cer1 were found to colocalize in the perinuclear endoplasmic reticulum, whereas Fum8 and Fum3 were found in endoplasmic reticulum-derived vesicles and the cytosol respectively [66]. The conversion of FB3 into FB1 away from the ceramide synthases is proposed as preventing self-toxification [66].

### Subcellular compartmentalization of terpenoid biosynthesis

#### Trichothecene biosynthesis

**Scheme 8:**
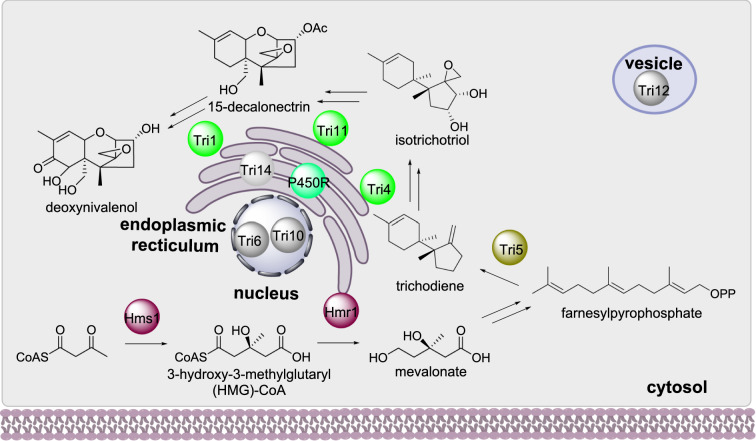
The sub-cellular biosynthesis of deoxynivalenol

Trichothecenes are sesquiterpene mycotoxins that causes toxicity in plants, animals, and humans, synthesized by *Fusarium* sp. and other fungi. The mevalonate pathway enzymes Hms1 and Hmr1 are involved in the biosynthesis of mevalonate which is a precursor to farnesylpyrophosphate (FPP). FPP is converted to trichodiene by the terpene cyclase Tri5 (Scheme 8) [67]. Trichodiene is then converted to deoxynivalenol (DON) by a series of enzymes including three P450 Tri1, Tri4, and Tri11 [68]. Tri14 is an enzyme with unknown function that is also required for DON biosynthesis [69].

The subcellular localization of several enzymes required for DON biosynthesis have been established using fluorescent protein fusions. The two P450s Tri1 and Tri 4 co-localize to structures termed “toxisomes” and the HMG-CoA reductase Hmr1 co-localizes with Tri4 upon induction of mycotoxin production [20]. The toxisomes were later established as being associated with the endoplasmic reticulum and include Tri14, the enzyme with unknown function. The terpene synthase Tri5 is localized in the cytosol [70] and the HMG-CoA synthase (Hms1) is also [71]. The major facilitator superfamily (MFS) protein Tri12p is located within vesicles [20].

The composition of the toxisome was further investigated using transmission electron microscopy (TEM) [70]. The P450s Tri1, Tri4, and Tri11 are anchored to the endoplasmic reticulum membrane with the P450 reductase (P450R) and Hmr1 [72]. Although the terpene cyclase Tri5 is established as being cytosolic, inactivation of this enzyme prevented toxisome assembly at the endoplasmic reticulum [72]. Tri5 therefore was proposed to interact directly with the toxisome complex or potentially to be post-translationally modified and attach itself to the endoplasmic reticulum membrane [72].

#### Gibberellin biosynthesis

**Scheme 9:**
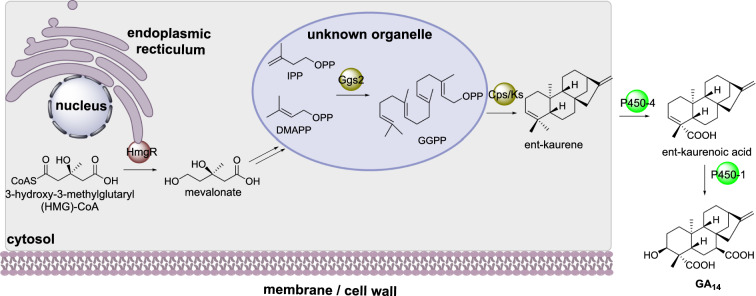
The partial sub-cellular biosynthesis of GA14. The location of the final oxidative transformations performed by the P450s are currently unknown

Gibberellic acids (GA) are a large family of diterpenoid compounds produced by fungi and plants that contain a gibberellan ring structure. In *Fusarium fujikuroi* the GGPS Ggs2 synthesizes GGPP which is converted to ent-kaurene by the diterpene cyclase Cps/Ks (Scheme 9) [73]. Two P450s, P450-4 and P450-1, convert ent-kaurene to ent-kaurenoic acid and GA14 respectively [74, 75], where GA14 is the precursor to the biologically active gibberellins used as plant phytohormones.

The subcellular localization of Ggs2, Csp/Ks, and HmgR were investigated using fluorescence protein fusion constructs. Cps/Ks was determined as having cytosolic localization but Ggs2 and HmgR were observed as small punctate structures [76]. Ggs2 contains a peroxisomal targeting signal but when co-expressed with a peroxisomal targeting fluorescence protein, the two did not co-localize, indicating that Ggs2 is contained within organelles that are not peroxisomes [76]. HmgR contains eight predicted transmembrane domains and its fluorescence pattern colocalized with the endoplasmic reticulum [76].

#### Paxilline biosynthesis

**Scheme 10:**
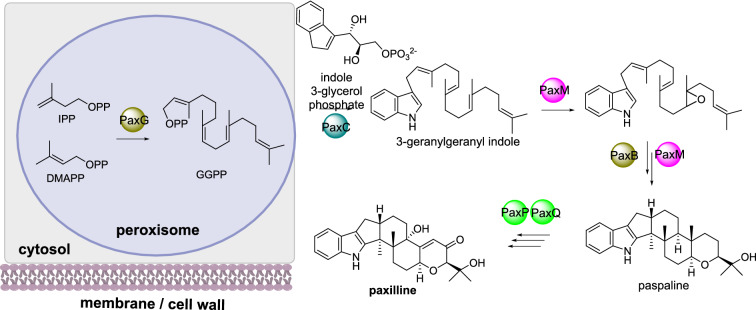
The sub-cellular biosynthesis of GGPP during the biosynthesis of paxilline. The localization of the remaining biosynthetic enzymes has not yet been established and therefore are drawn outside of the cartoon cell

Paxilline is an indole-diterpene produced by *Penicillium paxilli*. The cyclic diterpene core arises from geranylgeranyl diphosphate (GGPP) synthesized by PaxG, a geranylgeranylpyrophosphate synthase [77]. The prenyltransferase PaxC transfers GGPP to indole 3-glycerol phosphate to form 3-geranylgeranyl indole. In an unusual sequential manner PaxM, an FAD-dependent monooxygenase, and PaxB, a novel terpene cyclase [78, 79], form paspaline which is a common carbon skeleton observed in many fungal indole-diterpene metabolites (Scheme 10). Finally, two P450s, PaxP and PaxQ, covert paspaline to paxilline.

Localization studies using both N-terminal and C-terminal eGFP fusions led to unstable transformants [77]. Bioinformatics analysis of PaxG revealed a tripeptide sequence indicative of peroxisomal targeting. Fusing this tripeptide sequence to eGFP led to green fluorescence in punctate organelles that were determined to be peroxisomes [77]. When a truncated PaxG, lacking the tripeptide sequence, was used to complement the function of an inactivated PaxG, paxilline biosynthesis could not be restored, demonstrating that the enzyme needs to be localized to the correct subcellular compartment [77].

#### Aphidicolin biosynthesis

**Scheme 11:**
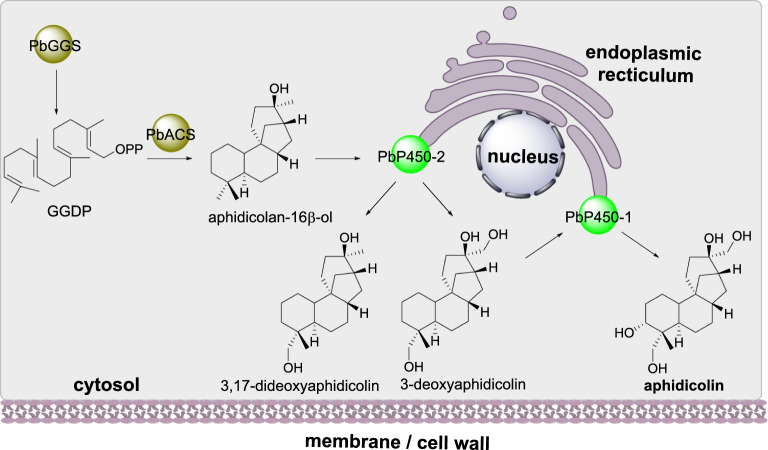
The sub-cellular localization of aphidicolin

Aphidicolin is a terpenoid that inhibits DNA polymerase [80]. In *Phoma betae* the biosynthesis of the terpene backbone arises via the geranylgeranyl diphosphate synthase (PbGGS) and the terpene synthase aphidicolin-16b-ol synthase (PbACS). Two P450s (PbP450-1 and PbP450-2) are required for two hydroxylations (Scheme 11) [81]. Investigation of aphidicolin biosynthesis using the heterologous host *Aspergillus oryzae* led to lower titers of production than expected so the subcellular localization of the individual biosynthetic enzymes were investigated in the heterologous host. Fusion of green fluorescence protein to each of the biosynthetic enzymes revealed that PbGGS and PbACS are localized to the cytoplasm whereas the two P450 enzymes are located at the endoplasmic reticulum membrane [82].

### Subcellular compartmentalization of meroterpenoid biosynthesis

#### Mycophenolic acid biosynthesis

**Scheme 12:**
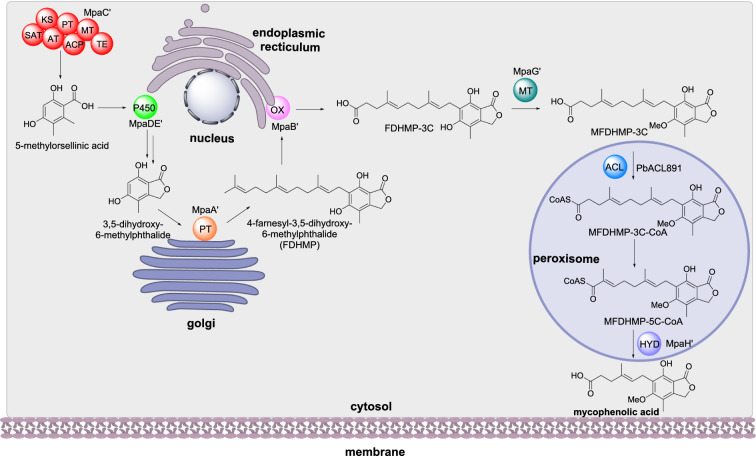
The sub-cellular biosynthesis of mycophenolic acid in *P. brevicompactum*

Mycophenolic acid is a meroterpenoid that inhibits inosine-5’-monophosphate dehydrogenase and is used commercially as an immunosuppressant drug [83, 84]. Recently, the biosynthesis of mycophenolic acid was established in *Penicillium brevicompactum* [85]. The NRPKS MpaC’ synthesizes 5-methylorsellinic acid, and the dual function P450/hydrolase MpaDE’ converts 5-methylorsellinic acid to 3,5-dihydroxy-7-(hydroxymethyl)-6-methylbenzoic acid and then 3,5-dihydroxy-6-methylphthalide. The prenyltransferase MpaA’ farnesylates 3,5-dihydroxy-6-methylphthalide to 4-farnesyl-3,5-dihydroxyphthalide (FDHMP) and the oxidase MpaB’ catalyzes the oxidative cleavage of FDHMP between the C19 and C20 double bond resulting in FDHMP-3 C. The O-methyltransferase MpaG’ methylates FDHMP-3 C to form MFDHMP-3 C. The acyl-CoA ligase PbACL891 converts MFDHMP-3 C to its acyl-CoA ester MFDHMP-3 C-CoA which is subsequently shortened via β-oxidation, controlled by the acyl-CoA hydrolase MpaH’, and finally released as mycophenolic acid (Scheme 12) [85].

MpaC’ and MpaG’ are reported as cytosolic enzymes and the subcellular localization of the membrane-associated enzymes was determined using N- or C-terminal GFP tags and specific organelle markers [85]. Both MpaB’ and MpaDE’ were detected localized with the endoplasmic reticulum while MpaA’ was determined to reside in ring like structures at the Golgi complex. MpaH’ contains a peroxisomal tripeptide sequence and, as expected, was found localized in peroxisomes; removal of the tripeptide sequence led to loss of localization [85].

### Subcellular compartmentalization of peptide biosynthesis

#### Non-ribosomal peptides

##### Penicillin biosynthesis

**Scheme 13:**
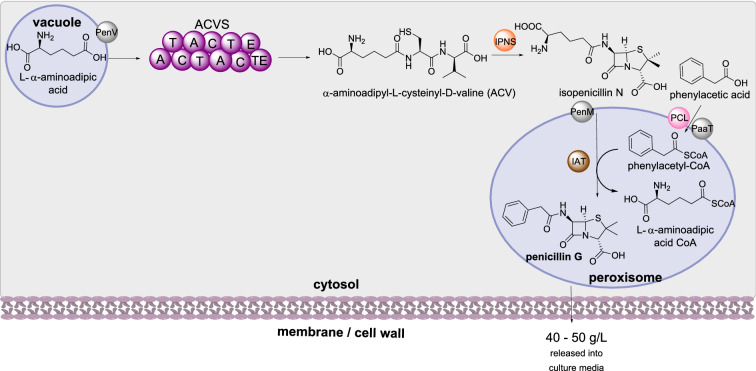
The sub-cellular biosynthesis of penicillin G. The mechanism of penicillin excretion is currently unknown

Penicillin is a commonly used antibiotic used to treat bacterial infections. Due to the importance of penicillin for human health, the sub-cellular localization of the biosynthetic enzymes for are some of the most well investigated (Scheme 13). In penicillin biosynthesis the NRPS (ACVS) selects, activates, and condenses L−α-aminoadipic acid, L-cysteine and L-valine to synthesize α-aminoadipyl-L-cysteinyl-D-valine (ACV), the first enzyme-free intermediate in the pathway [86]. Isopenicillin N synthase (IPNS) then converts ACV to isopenicillin N [87]. Isopenicillin N is then converted to penicillin G through the action of isopenicillin N acyltransferase (IAT) [88].

The sub-cellular localization of penicillin has been studied extensively. The non-proteinogenic amino acid L−α-aminoadipic acid is stored in vacuoles [89] and it is speculated that this a deliberate self-protection mechanism [90]. The localization of the NRPS ACVS has reported as being associated with small organelles [91] later identified as vacuoles [89], however ACVS has also been identified in the cytosol [92]. IPNS was detected in the cytosol and IAT was detected in small organelles with a diameter of 200–800 nm [91], later identified as peroxisomes [93]. Phenylacetyl-CoA ligase (PCL), required for converting phenylacetic acid into phenylacetyl-CoA, was identified as containing a peroxisomal signal peptide and later confirmed in the peroxisomes [93–95].

Three transporters have been identified for shuttling intermediates between compartments (Scheme 13). The first, PenV, transports L-α-aminoadipic acid from the vacuole to the cytoplasm and is located at the vacuolar membrane [96]. PenM transports isopenicillin N from the cytoplasm to peroxisomes; fusion of PenM with DsRed protein confirmed its peroxisomal location and over-expression using a strong promoter increased production of penicillin G [97]. PaaT transports phenylacetic acid from the cytoplasm to peroxisomes, preventing self-toxification [90]. Fusion of PaaT to DsRed combined with organelle markers and fluorescent laser scanning microscopy demonstrated localization in the peroxisomal membrane [98]. Furthermore, over-expression of PaaT lead to significantly increased titers of penicillin production.

The final transport of penicillin G from peroxisomes to outside of the cell and into the growth media proceeds *via* an unknown mechanism. It is interesting to note that isopenicillin N is also secreted into the culture medium by an unknown mechanism [99], and it is speculated that the penicillin biosynthetic pathway encodes two different metabolites, isopenicillin N and penicillin G, that possess different biological functions [90].

##### Cephalosporin C biosynthesis

**Scheme 14:**
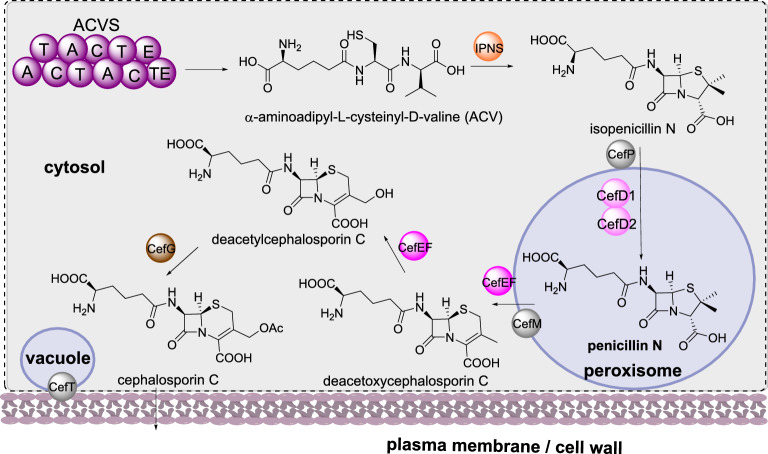
The sub-cellular biosynthesis of cephalosporin C. The localization of the remaining biosynthetic are inferred (shown in a dotted compartment) or have not yet been established and therefore are not attributed to a specific compartment

Cephalosporin C is an antibacterial agent produced by *Acremonium chrysogenum* that shares a similar biosynthesis to that of penicillin G, up to the formation of isopenicillin N (Scheme 14). In contrast to the penicillin pathway, the BGC encoding cephalosporin C contains eleven genes, split into two different clusters [90, 100]. The genes required for the early stages of biosynthesis (up to penicillin N) are located on one chromosome, however the genes encoding the later steps of the biosynthesis, *cefEF* and *cefG*, are located elsewhere on the genome [101]. In the divergent biosynthesis, isopenicillin N is epimerized to penicillin N by isopenicillin N-CoA ligase (CefD1) and isopenicillin N-CoA epimerase (CefD2), and then penicillin N is converted to deacetylcephalosporin C via deacetoxycephalosporin C (DAOC) by the bifunctional enzyme DAOC synthase / hydroxylase (CefEF) [102, 103]. In the final step deacetoxycephalosporin C is acetylated to form cephalosporin C [101].

There is less known about the sub-cellular localization of the biosynthetic enzymes that synthesize cephalosporin C compared to those required for penicillin biosynthesis. ACVS and IPNS are speculated to be cytosolic enzymes [90], analogous to the penicillin enzymes. CefD1 and CefD2 contain peroxisomal targeting sequences and homologs in *P. chrysogenum* were identified in the peroxisome [90, 95]. The transporters CefM and CefP also contain peroxisomal targeting sequences [95]; disruption of *cefM* led to an accumulation of penicillin N indicating a role in secretion from the peroxisomes [104], whereas disruption of *cefP* led to accumulation of isopenicillin N indicating a role in transporting isopenicillin N into the peroxisomes [105]. The sub-cellular localization of CefEF and CefG are speculated to be in the cytosol [90] and the final transporter CefT is indicated as being located both in the vacuoles and in the cell membrane, based on expression of CefT-GFP in the heterologous host *P. chrysogenum* [106]. Although inactivation of *cefT* did not prevent accumulation of cephalosporin C, overexpression led to almost twice as much production in both native and heterologous hosts [106, 107].

##### Fumiquinazoline biosynthesis

**Scheme 15:**
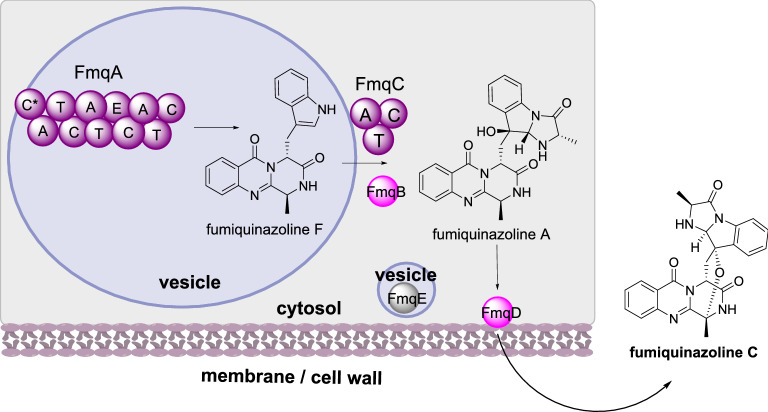
The sub-cellular biosynthesis of fumiquinazoline C. The final step of the biosynthesis occurs outside of the cell

Fumiquinazolines are a family of peptidyl alkaloids produced by various fungi that have cytotoxic properties [108]. The biosynthesis requires the trimodular NRPS (FmqA) that condenses anthranilate, alanine, and tryptophan to produce fumiquinazoline F [109] this is then oxidized by the FAD-dependent monooxygenase (FMO) FmqB and condensed with alanine through the action of the monomodular NRPS (FmqC) to synthesize fumiquinazoline A [110]. Finally, the FMO FmqD converts fumiquinazoline A to fumiquinazoline C (Scheme 15) [111]. Also encoded by the BGC is an MFS transporter, FmqE; the BGC is regulated by the conidiation-specific transcription factor BrlA that is not encoded within the BGC [112].

Investigations of metabolite accumulation determined that fumiquinazoline F and fumiquinazoline A are found in all fungal tissues at comparable levels but fumiquinazoline C accumulates in conidial tissues [112]. FmqA is located with vesicles that differ in size but are uniformly spaced within hyphae, but are not vacuoles, mitochondria, or nuclei. FmqB and FmqC were distributed evenly throughout the cytoplasm. A conserved signal motif was identified in FmqD indicating that this enzyme is secreted [111]. When this signal peptide was deleted fumiquinazoline A production increased whereas production of fumiquinazoline C decreased. Over-expression of FmqD fused to eGFP determined that FmqD is localized to the cell well and the extent of localization depends on the growth stage of the fungal cells [112]. Further investigations revealed that localization of FmqD is actin-dependent and requires endoplasmic reticulum-Golgi transport to the cell wall, as expected for secretory proteins. FmqE was also detected in vesicles within the hyphae but in a different arrangement to FmqA [112].

##### Cyclosporin biosynthesis

**Scheme 16:**
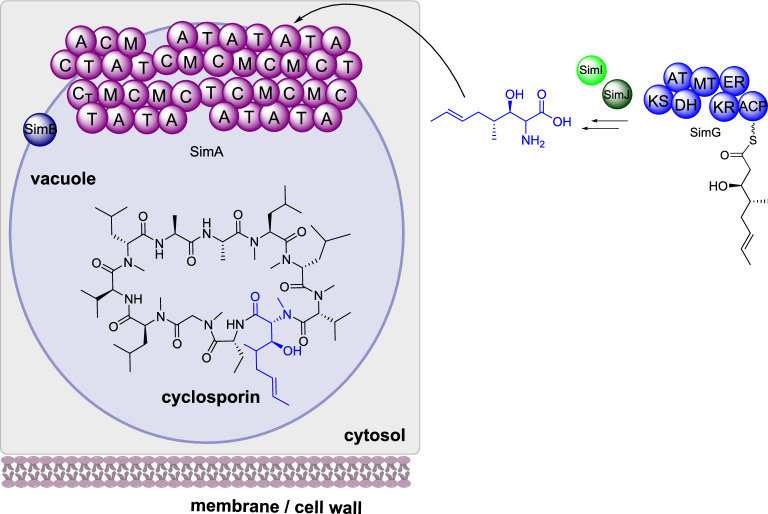
The partial sub-cellular biosynthesis of cyclosporin. Additional enzymes are required for the biosynthesis and are shown for completeness, however their location is unknown and so they are not drawn associated with any specific cellular compartment

Cyclosporin is a cyclic undecapeptide isolated from *Tolypocladium inflatum* that is used commercially as an immunosuppressant drug [113]. The biosynthetic gene cluster is proposed as consisting of 12 genes that encode: SimA, an 11 module NRPS; Sim B, an alanine racemase; SimE, a thioesterase; SimG, a highly reducing PKS; SimI, a P450; and SimJ, an aminotransferase [114]. SimG, SimI and SimJ collaborate to synthesize the unusual (4R)-4-[(E)-2-butenyl]-4-methyl-l-threonine and SimB racemizes D-alanine from L-alanine; these unnatural amino acids are incorporated by the NRPS SimA during biosynthesis and the terminal C domain releases the peptide by cyclization (Scheme 16). Also encoded in the gene cluster are: SimC, a cyclophilin; SimD, an ABC transporter; and SimL, a transcription factor [114].

The subcellular localization of SimA in *T. inflatum* was investigated using immunolocalization and electron microscopy. SimA and SimB were determined to be attached to the outside of the vacuolar membrane, in close proximity, and SimA was observed to exist as a globular complex consisting of 11 particles presumed to be the 11 amino-acid modules separated by linkers [115]. The final metabolite cyclosporin was found stored in high concentrations within vacuoles and can be released slowly *via* vacuolar / cytoplasmic membranes, or rapidly if the cell is lysed [115].

##### Triacetylfusarinine C and hydroxyferricrocin biosynthesis

**Scheme 17:**
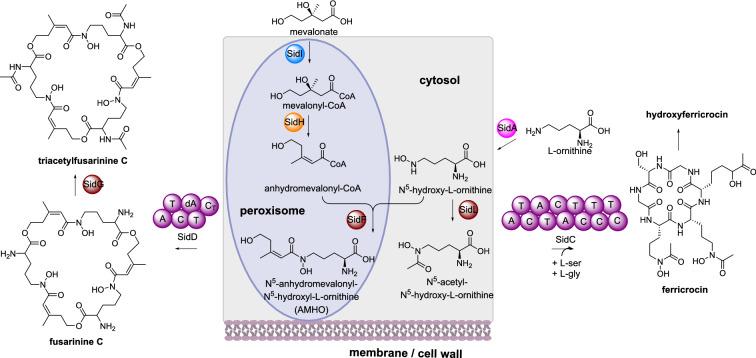
The partial sub-cellular biosynthesis of triacetylfusarinine C and hydroxyferricrocin. Additional enzymes required for biosynthesis are shown for completeness, however their location is unknown, therefore they are not shown inside the cartoon cell

Triacetylfusarinine C is a siderophore produced by various fungi that has an essential role in iron uptake and virulence [116]. Triacetylfusarinine C contains three N^2^-acetyl-N^5^-anhydromevalonyl-N^5^-hydroxyornithine residues joined through ester bonds. SidD is an iterative NRPS that synthesizes fusarinine C from N^5^-anhydromevalonyl-N^5^-hydroxyornithine (AMHO) [117]. AMHO is produced by: SidA, an ornithine monooxygenase; SidI, a mevalonyl-CoA ligase; SidH, a mevalonyl-CoA hydratase; and SidF, a anhydromevalonyl-CoA transferase [118, 119]. SidI and SidH synthesize anhydromevalonyl-CoA from mevalonate and SidF transfers this to N^5^-hydroxy-L-ornithine produced by SidA (Scheme 17). Finally, SidG, an acetyl transferase, catalyzes N^2^-acetylation of fusarinine C to produce triacetylfusarinine C.

Bioinformatic analysis of SidF, SidH, and SidI indicated C-termini peroxisomal targeting signals in SidF and SidH, and an N-terminal peroxisomal targeting signal in SidI [118]. Construction of GFP-tagged versions of the three enzymes in respective knock-out strains led to the detection of punctate structures observed in the cytoplasm and restoration of triacetylfusarinine C production. Peroxisomal location was confirmed though co-expression with peroxisomal targeting of red fluorescent protein. When the putative peroxisomal targeting signal was removed from SidH GFP was detected throughout the cytosol, however triacetylfusarinine C was not detected indicating that correct localization of SidH is required for biosynthesis [118]. The remaining biosynthetic enzymes are presumed to be localized in the cytosol but this is not known for certain. However, EstB, the esterase that hydrolyzes triacetylfusarinine C after binding iron, was detected in the cytoplasm of *A. fumigatus* particularly during iron starvation conditions [120].

A second siderophore pathway is also known in *A. fumigatus* where N^5^-hydroxy-L-ornithine is acetylated by the N-acetyltransferase SidL to generate N^5^-acetyl-N^5^-hydroxyornithine [121]. The NRPS SidC condenses N^5^-acetyl-N^5^-hydroxy-L-ornithine, serine and glycine to synthesize ferricrocin, which is hydroxylated to produce the siderophore hydroxyferricrocin (Scheme 17) [122]. eGFP fused SidL was used in complementation studies and epifluorescent microscopy determined that SidL is localized in the cytoplasm [121].

**Fig. 3:**
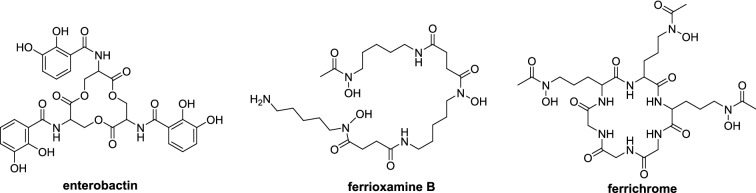
The structures of various siderophores investigated for their sub-cellular localization

The subcellular localization of siderophore transporters has been investigated in *Saccharomyces cerevisiae* using GFP fusion proteins. The structures of the respective siderophores are shown in Fig. 3. Enb1 is an enterobactin transporter that is targeted to the plasma membrane whereas Sit1 is a ferrioxamine B transporter that is targeted to the vacuolar degradation pathway when no substrate is present [123]. Similarly, Arn1p is a transporter for the uptake of ferrichrome that is sorted from the trans-Golgi network to the vacuolar lumen when no siderophore is present [124].

#### Ribosomally synthesized peptides

##### Amanitin biosynthesis

**Fig. 4:**
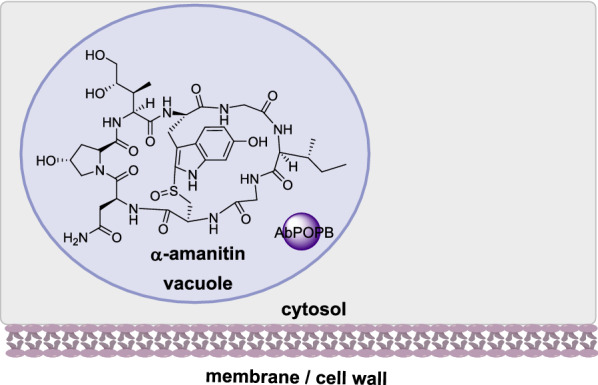
The sub-cellular localization of AbPOPB in a-amanitin biosynthesis

Amatoxins are ribosomally synthesized and post-translationally modified peptides (RiPPs) produced by select mushroom species that are responsible for severe mushroom poisonings [125]. Amatoxins consist of eight peptide residues that are cyclized and contain tryptathione, sulfoxide, and hydroxyl functionalities, and the biological target is RNA polymerase II [126]. Two genes have been characterized in *Amanita bisporigera* that are required for amanitin biosynthesis (Fig. 4): AMA1 that encodes a propeptide of 35 amino acids [127] and AbPOPB that encodes a prolyl oligopeptidase that cleaves the proprotein (Luo et al., 2010). The additional enzymes required for post-translational modification of the core peptide are unknown [126].

The role of AbPOPB was confirmed from a distantly related mushroom *Galerina marginate*; recombinant GmPOPB was shown to catalyze hydrolysis and cyclization, two non-processive reactions in amanitin biosynthesis [128, 129]. To determine the subcellular localization of amanitin and POPB in *A. bisporigera*, immunolocalization techniques were used that indicate that amanitin and AbPOPB colocalize in vacuoles [130].

## Enzyme trafficking and self-resistance to toxic intermediates

### Transient recruitment of enzymes and trafficking

An emerging theme in several of the examples discussed above is the difficulties in precisely locating various biosynthetic enzymes, due to conducting experiments at different stages of fungal development, or conflicting results between bioinformatics predictions and microscopic/chemical observations. Subcellular localization of biosynthetic enzymes therefore appears to be a highly dynamic process and requires trafficking of various enzymes to specific locations.

For example, in *A. fumigatus* the four early-stage enzymes required for melanin biosynthesis are found localized in endosomes but lack signal peptide or transmembrane domains, and the two-late stage enzymes are found at the cell wall. Subsequent experiments using size-exclusion chromatography and coimmunoprecipitation assays determined several examples of protein-protein interactions and complex formation between the early- and late-stage enzymes [131]. However, it was not the formation of an enzyme complex that was responsible for the enzyme trafficking between sub-cellular locations; instead, post-translational lipid modification appears to be the major mechanism. By using a combination of chemical reporters and liquid chromatography – tandem mass spectrometry, three early-stage melanin enzymes, Alb1, Ayg1 and Arp2, were discovered to be palmitoylated [131]. When *A. fumigatus* Alb1-eGFP fusion strains were grown in the presence of the palmitoylation inhibitor 2-bromopalmitate, Alb1 was no longer localized and instead was observed diffused in the cytoplasm [131].

Melanin biosynthesis in *B. cinerea* is also proposed to involve enzyme trafficking since the subcellular localization of BcBRN1/2 is observed in the cytoplasm, endosomes, and the cell wall [46]. This correlates well with the observed toxicity of scytalone and the requirement of BcBRN1/2 in its synthesis and subsequent conversion (Scheme 3C).

Similar trafficking mechanisms have been observed or proposed in the biosynthesis of aflatoxin, melanin, patulin, penicillin, cephalosporin, and fumiquinazoline. The dynamic nature of protein translocation and subsequent trafficking to the target organelle may explain the difficulties of accurate bioinformatic predictions of some biosynthetic enzymes. However, this strategy of localizing specific biosynthetic enzymes and intermediates to different organelles enables some control over confining toxic intermediates and explains how intermediates are transported to subsequent organelles. Ultimately, fusion of different organelles leads to larger organelles, known as toxisomes, that contain intricate enzyme complexes and accumulate the increasingly toxic intermediates, thereby protecting the cell from deleterious molecules [18, 132].

### Alternative mechanisms enabling self-resistance to toxic intermediates

It is well known that many fungal BGCs contain transporters which are proposed to provide fungi mechanisms to protect themselves, reviewed elsewhere [133]. Alternatively, BGCs may include an additional copy of the target protein that acts as a decoy, capturing the toxic specialized metabolite and preventing disastrous effects, also reviewed elsewhere [134]. However, for BGCs that encode neither transporters nor self-resistance proteins, other mechanisms must be available such as detoxifying enzymes, as first reported in gliotoxin biosynthesis [135].

**Scheme 18:**
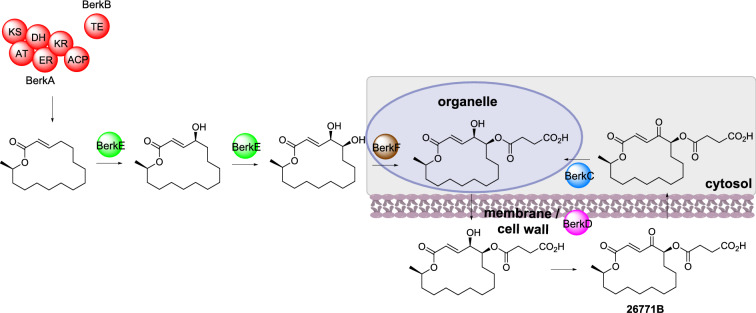
The partial sub-cellular biosynthesis of A26771B. The location of the HRPKS, TE and P450 were not reported, therefore they are not drawn within the cartoon cell

Recently, a dual compartmentalized / detoxification enzyme strategy for self-resistance was reported during the biosynthesis of A26771B [136]. A26771B is a 16-membered macrolide antibiotic produced by *Penicillium egyptiacum* (Scheme 18). The BGC contains a HRPKS (BerkA), a trans-acting thiolesterase (TE; BerkB), a short-chain reductase/dehydrogenase (SDR; BerkC), a flavin-dependent monooxygenase (FMO; BerkD), a P450 (BerkE), and an acetyltransferase, (BerkF). The role of each enzyme was established through heterologous gene expression in *Aspergillus nidulans*, however co-expression of *berkABCDEF* lead to production of the inactive precursor; when *berkC* was omitted these precursors were no longer evident. The role of BerkC was established as reducing A26771B to the inactive form; BerkC therefore appears to function as a detoxification enzyme, inactivating the toxicity of A26771B inside *P. egyptiacum* [136].

The sub-cellular localization of BerkC and BerkD were investigated by individually fusing to super folder GFP (sfGFP) and expressing in *A. nidulans* [136]. BerkC was detected in the cytoplasm whereas BerkD was localized to the cell wall and septa, indicating that it is secreted extracellularly. The location of BerkF was also investigated through fusion with sfGFP and determined to be localized to intracellular organelles. It is proposed that the inactive precursor is exported out of the cell and the extracellular BerkD oxidizes this intermediate to the active A26771B [136]. As A26771B can be transported back into the cell, BerkC reduces A26771B to its inactive form which is exported outside of the cell in a redox cycle. It is currently unknown how this transport occurs, but it is proposed that BerkF and / or the acyl side chain introduced by BerkF facilitate the export [136].

The trafficking of redox enzymes to the cell wall / outside of the cell has been reported in the biosynthesis of melanin [19, 46], patulin [53], and fumiquinazoline [112], perhaps indicating a frequent self-resistance mechanism. Finally, the containment of specialized metabolites has been also reported within specific tissues e.g. hyphae and conidia [137], and spores [112, 132, 138].

## Conclusions

This review collates examples of fungal specialized metabolite biosynthetic pathways where the subcellular localization of all, or some, of the biosynthetic pathways have been elucidated, revealing the complexity of these studies. Many pathways and associated enzymes are often individualized with the same enzyme classes being in different subcellular compartments in different fungi, or even the same metabolite being in different subcellular compartments in different fungi. Furthermore, subcellular localization of biosynthetic enzymes, intermediate trafficking, and transport is highly dynamic, demonstrating the challenges facing researchers studying this phenomenon.

To date single biosynthetic pathways are studied at a given time but as more investigations into these systems are conducted it will be interesting to observe how competing biosynthetic pathways are contained, or separated, within a cell. Future directions will need to uncover the strength, timescales, and kinetics of protein-protein interactions which in turn may require novel chemical and biophysical methods. However, this knowledge is crucial for metabolic engineers and for establishing new and robust heterologous hosts to generate larger-scale production of medicinally and agriculturally important fungal molecules.

## Data Availability

Not applicable.

## Abbreviations

BGC: Biosynthetic gene clusters
CS: Ceramide synthase
CYC: Cyclase
DH: Dehydratase
DMAPP: Dimethylallyl pyrophosphate
DON: Deoxynivalenol
EDA: 9,10-Epoxy-8-hydroxy-9-methyl-decatrienoic acid
eGFP: Enhanced green fluorescence protein
FAS: Fatty acid synthase
FMO: Flavin-dependent monooxygenase
FPP: Farnesylpyrophosphate
GGPS: Geranylgeranylphosphate synthase
HMG-CoA: β-Hydroxy-β-methylglutaryl-CoA
HMGS: β-Hydroxy-β-methylglutaryl synthase
HRPKS: Highly reducing polyketide synthase
HYD: Hydrolase
IPP: Isopentenyl pyrophosphate
Lac: Laccase
MFS: Major facilitator superfamily
MT: Methyltransferase
NRPKS: Non-reducing polyketide synthase
NRPS: Non-ribosomal peptide synthetase
OX: Oxidase
OXR: Oxidoreductase
P450: Cytochrome P450 monooxygenase
PKS: Polyketide synthases
PKS/NRPS: Polyketide synthase / non-ribosomal peptide synthetase
PRPKS: Partially reducing polyketide synthase
PT: Prenyltransferase
Red: Reductase
RiPP: Ribosomally synthesized and post-translationally modified peptide
TC / TS: Terpene cyclase / terpene synthase
TEM: Transmission electron microscopy
TF: Transcription factor
THN: 1,3,6,8-Tetrahydroxynaphthalene
Trans: Transporter
U: Unknown / hypothetical protein
UV: Ultra-violet
